# Smoking, Habitual Tea Drinking and Metabolic Syndrome in Elderly Men Living in Rural Community: The Tianliao Old People (TOP) Study 02

**DOI:** 10.1371/journal.pone.0038874

**Published:** 2012-06-14

**Authors:** Chin-Sung Chang, Yin-Fan Chang, Ping-Yen Liu, Chuan-Yu Chen, Yau-Sheng Tsai, Chih-Hsing Wu

**Affiliations:** 1 Department of Family Medicine, National Cheng Kung University Hospital, Tainan, Taiwan; 2 Department of Internal Medicine, National Cheng Kung University Hospital, Tainan, Taiwan; 3 Tianliao District Public Health Center, Kaohsiung City, Taiwan; 4 Graduate Institute of Clinical Medicine, College of Medicine, National Cheng Kung University, Tainan, Taiwan; Tehran University of Medical Sciences, Iran (Islamic Republic of)

## Abstract

The literature shows an inconsistent relationship between lifestyle behaviors and metabolic syndrome (MetS), especially in the elderly. We designed this study to investigate the interrelationships among cigarette smoking, tea drinking and MetS, and to verify the factors associated with MetS in elderly males dwelling in rural community. In July 2010, with a whole community sampling method, 414 male subjects aged over 65 dwelling in Tianliao township were randomly sampled. The response rate was 60.8%. Each subject completed the structured questionnaires including sociodemographic characteristics, habitual behaviors (including cigarette smoking and tea drinking habits) and medical history. After an overnight fast, the laboratory and anthropometric data were obtained. MetS was confirmed according to the criteria defined by the modified NCEP ATP III for the male Chinese population. Subjects were split into either non-MetS or MetS groups for further analysis. Of the 361 subjects with complete data, 132 (36.6%) elderly men were classified as having MetS. Using binary logistic regression, body mass index, serum uric acid, high sensitivity C-reactive protein, HOMA index, current smokers (OR = 2.72, 95%CI: 1.03 ∼ 7.19), total smoking amount > = 30 (OR = 2.78, 95%CI: 1.31 ∼ 5.90) and more than 20 cigarettes daily (OR = 2.54, 95%CI: 1.24 ∼ 5.18) were positively associated with MetS. Current un- or partial fermented tea drinker (OR = 0.42, 95%CI: 0.22 ∼ 0.84), tea drinking habit for 1–9 years (OR = 0.36, 95%CI: 0.15 ∼ 0.90) and more than 240cc daily (OR = 0.35, 95%CI: 0.17 ∼ 0.72) were negatively associated with MetS. In conclusion, this study suggests that smoking habit was positively associated with MetS, but tea drinking habit was negatively associated with MetS in elderly men dwelling in rural community.

## Introduction

Metabolic syndrome (MetS) has become a major health hazard due to its increasing prevalence worldwide [1], [2]. The diagnostic criteria of MetS are a combination of clinical features, including central obesity, glucose intolerance, high blood pressure and dyslipidemia [3], and the condition is an important predictor for diabetes, cardiovascular disease (CVD) and all cause mortality [4]. Early detection of the associated factors will enhance preventive strategies in the preclinical stage of MetS.

The relationship between lifestyle behaviors and MetS has recently attracted more attention [5]–[7], especially the relationship between cigarette smoking and MetS [7]–[9]. However, the results are inconclusive which may be due to the different parameters of smoking habits used either by current smoking status, current smoking amount or the total smoking amount (TSA, pack-years) [7], [8]. In addition, evidence suggests that habitual tea consumption may significantly reduce the risk of developing hypertension and have an inverse relationship with body fat distribution and hyperglycemia [10]–[12]. Therefore, the effect of habitual tea consumption on MetS is worth further investigation. To the best of our knowledge, there are few studies that focus on the effects of lifelong TSA on MetS, and no studies have examined the association between tea consumption and MetS in the elderly. The aim of this study was therefore to investigate the interrelationships among the cigarette smoking habits (with different parameters), habitual tea consumption and MetS. We also aimed to verify the factors associated with MetS for the elderly males dwelling in a rural community.

## Materials and Methods

### Study Population

Tianliao township, with 23.7% of its residents aged over 65, is a rural community located in Kaohsiung County, southern Taiwan, containing only one primary care unit. The Tianliao Old People (TOP) study has been conducted in an aged cohort since 2009 in order to develop better community-oriented primary care (COPC) services. According to the 2010 census registered data, there were 1,033 elderly men aged over 65 in Tianliao township. An epidemiological survey using whole community sampling method was performed in July 2010. After excluding empty houses (n = 269), death (n = 21) and non-ambulatory subjects (n = 62), only 681 subjects were eligible and 414 subjects were enrolled in the study. The response rate was 60.8% and the statistical power was 0.80. There were no statistically significant differences in mean age (74.6±6.1 vs 75.3±7.5, *p* = 0.17) and age distribution between responders and non-responders (*p*>0.05). Finally, a total of 361 ambulatory men aged 65 to 98 with completed data were enrolled for the final analysis.

This study was approved by the Institute Review Board of National Cheng Kung University Hospital (IRB no: ER-99-111) and each subject signed the inform consent before examination.

### Data Collection

#### Questionnaires

Each subject was interviewed by well-trained research assistants and completed 20-minute structured questionnaires [10], [11], [13]. The questionnaires involved several aspects: (a) sociodemographic characteristics, including age, and marital, occupational and educational status; (b) habitual behaviors, including physical activity, cigarette smoking, coffee, alcohol, and tea drinking; and (c) medical history, including history of diabetes mellitus (DM), hypertension (HTN), hyperlipidemia, thyroid disease, cancer, and so on.

Habitual tea, alcohol or coffee drinking was defined as those who consumed tea, alcohol or coffee more than once a week for more than half a year. The questionnaire for tea consumption included current drinking habit, tea types, duration and daily amount of tea. The question were “Have you drunk tea habitually once a week for at least 6 months?” Subjects who answered “yes” were coded as habitual tea drinkers and completed the following questions. 1) Have you drunk tea habitually now? 2) How many years have you been drinking tea in this way? 3) What kind of tea (green, black, oolong or puerh) was mostly consumed? 4) How often do you drink tea each week or day? 5) How much (milliliters) tea do you drink each day? Finally, the average amount of daily tea consumption (milliliters) was calculated [(days x volume of tea extracts each day)] [10], [11]. Current tea consumers drank different kinds of teas including green tea (14.5%), black tea (34.5%), oolong tea (57%), puerh tea (2%) and others (3%) with 7% tea consumers drank more than one kind of teas. According to the preparation process of teas, they were classified as fermented tea (black tea or puerh tea) and un- or partial fermented tea (green tea or oolong tea) for further analysis. As the mean duration and the daily amount of tea drinking of our subjects was nearly 10 years and 240 cc daily, they were chosen as the cutoff point for further discussing the time effect of habitual tea consumption on MetS.

The subjects’ smoking habits were obtained from the questionnaire, including current smoking habits, history of quitting smoking, duration of smoking habit and average daily cigarette consumption. A current smoker was defined as a subject who had smoked more than 100 cigarettes and was still smoking [13]. An ex-smoker was defined as having stopped smoking for more than half a year [13]. Assuming 20 cigarettes per pack, total smoking amount (TSA,pack-years) was estimated using the following formula: 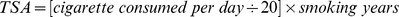. As the mean of TSA and daily smoking amount in all smokers was nearly 30 pack-years and 20 cigarettes daily, it was chosen as the cutoff point in defining non-smoker (TSA = 0, daily smoking amount = 0), mild to moderate (TSA<30, daily smoking amount<20) and heavy smoker (TSA≥30, daily smoking amount≥20) to examine the effects of smoking habits on MetS.

Marital status was defined as ‘coupled’ if the subject was married and still lived with their partner [14]. Because the average educational status was relatively low, subjects were categorized as literate or illiterate for the analysis. The total physical activity score of the participants was calculated using the short form of the International Physical Activity Questionnaire (IPAQ) [15] and was categorized by tertiles (low, middle and high level) for further analysis [16].

#### Anthropometric parameters

With the subject wearing light clothing without shoes, body weight (BW, to the nearest 0.1 kg, DETECTO™) and body height (BH, to the nearest mm, DETECTO™) were all measured and the body mass index (BMI, kg/m^2^) was then calculated. Standing naturally, looking forward and wearing only their underwear, the subjects’ waist circumferences (WC) were measured midway between the lateral lower rib margin and the superior anterior iliac crest with a standard tape (Gulick II®, WI, USA, to the nearest mm) at the end of a gentle expiration phase [11] by a same trained staff.

#### Diagnosis of metabolic syndrome

After overnight fasting, venous blood was obtained for the biochemistry parameters including fasting glucose, high density lipoprotein cholesterol (HDL-C), triglyceride, uric acid (UA), fasting insulin, and high sensitivity C-reactive protein (hs-CRP) [17]. HOMA index was obtained by fasting glucose times fasting insulin divided by 405 [18]. Metabolic syndrome was confirmed if a subject met three or more of the following five criteria defined by the National Cholesterol Education Program Adult Treatment Panel III Guideline and modified by the International Diabetes Federation specifically for the male Chinese population, including (a) fasting plasma glucose level ≥100 mg/dL or with hypoglycemic agent, (b) serum HDL-C level <40 mg/dL or with lipid lowering medication, (c) serum triglyceride level ≥150 mg/dL or with lipid lowering medication, (d) systolic blood pressure ≥130 mmHg or diastolic blood pressure ≥85 mmHg or with anti-hypertension medication, and (e) WC ≥90 cm [3], [19].

### Data Analyses and Statistical Methods

All continuous variables, including age, BMI, WC, TSA and laboratory data, were expressed as means (SD). The dichotomous data, such as socio-demographic data, habitual behaviors including alcohol drinking, tea consumption habit, different smoking habits and MetS, were expressed as percentages. Data with missing values were excluded when analyzed. The authors used a log-transformation method for the HOMA index and hs-CRP to fit the normal distribution model. Binary logistic regression analysis was used to assess the independent contribution to MetS by possibly associated factors that shows statistical significance in univariate analysis or had been emphasized in previous studies, including age, BMI, UA, log HOMA index, log hs-CRP, occupational status, marital status, educational status, alcohol and coffee drinking habits, tea consumption habit, smoking habit and physical activity. To evaluate the independent effect of smoking and tea drinking habits on MetS, three logistic regression models were formulated. In Model I, the current effects of smoking status (including non-smoker, ex-smoker and current smoker) and different types of tea drinking habits on MetS was analyzed. In Model II, the cumulative effects of smoking habit (including non-smoker, mild to moderate and heavy smoker defined by TSA) and tea drinking habit (grouped by less than or over 10 years) was analyzed. In Model III, the daily consumption of cigarette smoking habit (including non-smoker, mild to moderate and heavy smoker defined by daily smoking amount) and tea drinking habit (including non-current tea drinker, mild to moderate and heavy tea drinker defined by daily tea consumption) will be analyzed subsequently. All analyses were performed using the Statistical Package of Social Science for Windows software Version 16 (SPSSWIN, version 16.0, Chicago, USA). Statistical significance was defined as *p*<0.05 for two-tailed analysis.

## Results

The average age of the 361 subjects was 74.7±6.1. The mean WC and BMI were 86.4±10.6 cm and 24.1±3.1 kg/m^2^, respectively. One hundred and thirty-two (36.6%) of the subjects met the MetS diagnostic criteria. Compared with the non-MetS subjects, those with MetS had relatively higher BMI, WC, heavy smoker defined by TSA, UA and HOMA indices, but relatively lower levels of physical activity (Table 1). The interrelationships between smoking, tea drinking habits and all major associated factors were analyzed and showed no or relatively small correlation (Table 2 and Table 3).

**Table 1:** Demographic and Laboratory Data for the Without (MetS(−)) and With (MetS(+)) Metabolic Syndrome Groups of Elderly Males Living in a Rural Community (N = 361).

		MetS(−)	MetS(+)	*P*
Number		229 (63.4)	132 (36.6)	
Age (years)		74.6 (6.2)	74.9 (5.9)	0.69
Body mass index (kg/m^2^)		22.9 (2.8)	26.1 (2.6)	<0.001
Waist circumference (cm)		82.3 (10.2)	93.4 (6.9)	<0.001
Uric Acid (mg/dl)		6.4 (1.3)	7.3 (1.6)	<0.001
HOMA index		1.3 (1.0)	3.0 (3.5)	<0.001
hsCRP (mg/L)		2.8 (4.8)	3.6 (6.5)	0.14
Total smoking amount (pack-years)		27.9 (38.3)	33.8 (42.8)	0.18
Central obesity (≥90 cm) #	Yes	42 (18.3)	99 (75.0)	<0.001
Elevated blood pressure#	Yes	166 (72.5)	120 (90.9)	<0.001
Impair fasting glucose#	Yes	63 (27.5)	102 (77.3)	<0.001
Hypertriglyceridemia#	Yes	10 (4.4)	75 (56.8)	<0.001
Decreased HDLC#	Yes	24 (10.5)	89 (67.4)	<0.001
Occupational status	Yes	116 (50.7)	53 (40.2)	0.05
Lived with partner	Yes	188 (82.1)	107 (81.1)	0.81
Literate	Yes	31 (13.5)	21 (15.9)	0.54
Alcohol drinking	Yes	82 (35.8)	49 (37.1)	0.80
Coffee drinking	Yes	18 (7.9)	8 (6.1)	0.52
Current tea drinking				0.69
Non-current tea drinking		97 (42.4)	62 (47.0)	
0<Tea drinking years<10		31 (13.5)	17 (12.9)	
Tea drinking years≥10		101 (44.1)	53 (40.1)	
Kinds of tea drinking				0.11
Fermented tea		37(28.0)	29(41.4)	
*Unfermented tea		95(72.0)	41(58.6)	
Smoking				<0.05
Pack-years = 0		88 (38.4)	49 (37.1)	
0<Pack-years<30		64 (27.9)	20 (15.2)	
Pack-years≥30		77 (33.6)	63 (47.7)	
Physical activity (IPAQ-short form)				<0.05
Low		62 (27.1)	58 (44.0)	
Middle		84 (36.7)	37 (28.0)	
High		83 (36.2)	37 (28.0)	

Continuous data were analyzed with independent-sample T test; dichotomous data were analyzed with chi-square test.

HDLC: high density lipoprotein cholesterol; HOMA: Homeostatic model assessment;

hsCPR: high sensitivity C-reactive protein;

IPAQ: International Physical Activity Questionnaire.

data expressed as: number (percent), mean (standard deviation).

# modified ATP III definition of metabolic syndrome.

* Unfermented tea including green tea (unfermented) and oolong tea (partial fermented).

**Table 2:** Correlation Coefficients for Tea Drinking Habits and Associated Factors in Elderly Males Living in a Rural Community.

		Current tea drinking	Habitual tea consumption	Daily tea consumption
Age		−0.098	−0.080	−0.050
Body mass index (kg/m^2^)		0.018	0.015	0.002
Uric acid (mg/dl)		0.027	0.068	0.041
HOMA index#		0.027	0.012	0.029
hsCRP (mg/L)#		0.041	0.018	0.013
Occupational status	(No = 0, Yes = 1)	−0.026	0.008	−0.005
Lived with partner	(No = 0, Yes = 1)	0.038	0.031	0.041
Literate	(No = 0, Yes = 1)	0.038	0.032	0.048
Alcohol drinking	(No = 0, Yes = 1)	0.136^a^	0.178^a^	0.133^a^
Coffee drinking	(No = 0, Yes = 1)	0.162^a^	0.108^a^	0.103
Current smoking habit		0.171^a^	–	–
Total smoking amount		–	0.258^b^	–
Daily smoking amount		–	–	0.257^b^
Physical activity (IPAQ-short form)		−0.041	−0.015	−0.041

a *P*<0.05; ^b^ *P*<0.001. Data were analyzed with pearson correlation analysis.

HOMA: Homeostatic model assessment; hsCPR: high sensitivity C-reactive protein;

IPAQ: International Physical Activity Questionnaire.

# :Log transformation.

* Unfermented tea including green tea (unfermented) and oolong tea (partial fermented).

Current tea drinking : (No = 0, Fermented = 1, Unfermented = 2).

Habitual tea consumption : (Non-current tea drinking = 0, Tea drinking years<10 = 1, Tea drinking years≥10 = 2).

Daily tea consumption: (Non-current tea drinking = 0, Daily amount<240 cc = 1, Daily amount≥240 cc = 2).

Current smoking habit (Non-smoker = 0, Ex-smoker = 1, Current smoker = 2).

Total smoking amount (Non-smoker = 0, 0<Pack-years<30 = 1, Pack-years≥30 = 2).

Daily smoking amount (Non-smoker = 0, Daily amount<20 = 1, Daily amount ≥20 = 2).

Physical activity (IPAQ-short form) (Low = 0, Middle = 1, High = 2).

**Table 3:** Correlation Coefficients for Smoking Habits and Associated Factors in Elderly Males Living in a Rural Community.

		Current smoking habit	Total smoking amount	Daily smoking amount
Age		−0.012	0.044	0.011
Body mass index (kg/m^2^)		−0.085	−0.027	−0.022
Uric acid (mg/dl)		−0.018	0.025	0.042
HOMA index#		−0.101	−0.067	−0.060
hsCRP (mg/L)#		0.004	0.026	0.009
Occupational status	(No = 0, Yes = 1)	0.023	−0.041	−0.037
Lived with partner	(No = 0, Yes = 1)	−0.115^a^	−0.167^a^	−0.135^a^
Literate	(No = 0, Yes = 1)	−0.069	0.049	−0.022
Alcohol drinking	(No = 0, Yes = 1)	0.283^b^	0.289^b^	0.307^b^
Coffee drinking	(No = 0, Yes = 1)	0.031	−0.015	−0.003
Current tea drinking		0.171^a^	–	–
Habitual tea consumption		–	0.258^b^	–
Daily tea consumption		–	–	0.257^b^
Physical activity (IPAQ-short form)		−0.005	−0.093	−0.080

a *P*<0.05; ^b^ *P*<0.001. Data were analyzed with pearson correlation analysis.

HOMA: Homeostatic model assessment; hsCPR: high sensitivity C-reactive protein;

IPAQ: International Physical Activity Questionnaire.

# :Log transformation.

* Unfermented tea including green tea (unfermented) and oolong tea (partial fermented).

Current tea drinking : (No = 0, Fermented = 1, Unfermented = 2).

Habitual tea consumption : (Non-current tea drinking = 0, Tea drinking years<10 = 1, Tea drinking years≥10 = 2).

Daily tea consumption: (Non-current tea drinking = 0, Daily amount<240 cc = 1, Daily amount≥240 cc = 2).

Current smoking habit (Non-smoker = 0, Ex-smoker = 1, Current smoker = 2).

Total smoking amount (Non-smoker = 0, 0<Pack-years<30 = 1, Pack-years≥30 = 2).

Daily smoking amount (Non-smoker = 0, Daily amount<20 = 1, Daily amount ≥20 = 2).

Physical activity (IPAQ-short form) (Low = 0, Middle = 1, High = 2).

Using a binary logistic regression method, three models were analyzed consecutively to evaluate the interrelationships among smoking habit, tea drinking habit, associated factors and MetS. The Hosmer-Lemeshow goodness-of-fit test of the three models revealed reasonable model fit (*p*>0.05), and the Nagelkerke and Cox & Snell R squares were calculated. All these three models revealed that BMI, UA and HOMA index are significantly associated factors for MetS in the elderly men living in rural community. Model I focused on the current effects of smoking status and different types of tea drinking habits. In model I, after adjustment for age, BMI, UA, HOMA index, hs CRP, physical activity, psycho-social factors (occupational status, marital status, educational status), alcohol and coffee drinking habits, the current tea drinker who consumed un- or partial fermented tea habitually had significant negative whilst the current smoker had positive association with MetS compared with those who were non- tea drinker or non-smoker (Table 4). Model II focus on the cumulative effects of smoking and tea drinking habits, represented by the total smoking amount and duration of habitual tea consumption. In model II, MetS showed the significant positive association with total smoking amount (pack-years≥30 vs. non-smoker), but negative association with the duration of habitual tea drinking (tea drinking years<10 vs. no current tea drinking habit) (Table 5). Model III focus on the daily consumption of cigarette smoking and tea drinking. Compared with non-smoker and non-habitual tea drinker, current smoker with more than 20 cigarettes daily had positive association and current tea drinker with more than 240 cc daily had negative association with MetS (Table 6).

**Table 4:** Binary Logistic Regression Model for Associated Factors (Focus on Current Smoking Status and Different types of Tea Drinking Habits) of Metabolic Syndrome in Elderly Males Living in a Rural Community (N = 361).

					Odds ratio	95%CI		*P*
Age					1.02	0.97,	1.08	0.45
Body mass index (kg/m^2^)					1.38	1.21,	1.60	<0.001
Uric acid (mg/dl)					1.44	1.16,	1.80	0.001
HOMA index#					4.52	2.54,	8.05	<0.001
hsCRP (mg/L)#					1.22	0.94,	1.59	0.13
Occupational status			(No = 0, Yes = 1)		0.74	0.37,	1.47	0.39
Lived with partner			(No = 0, Yes = 1)		1.49	0.67,	3.33	0.33
Literate			(No = 0, Yes = 1)		0.87	0.39,	1.96	0.74
Alcohol drinking			(No = 0, Yes = 1)		1.23	0.65,	2.32	0.52
Coffee drinking			(No = 0, Yes = 1)		0.92	0.27,	3.14	0.90
Current tea drinking								
			(No = 0)		1			
			(Fermented = 1)		0.64	0.29	1.42	0.27
			*(Unfermented = 2)		0.42	0.22	0.84	0.01
Current smoking habit								
		(Non-smoker = 0)			1			
		(Ex-smoker = 1)			1.68	0.84,	3.33	0.14
	(Current smoker = 2)				2.72	1.03,	7.19	0.04
Physical activity (IPAQ-short form)								
				(Low = 0)	1			
				(Middle = 1)	0.73	0.35,	1.53	0.40
				(High = 2)	0.77	0.34,	1.74	0.53

HOMA: Homeostatic model assessment; hsCPR: high sensitivity C-reactive protein;

IPAQ: International Physical Activity Questionnaire.

Dependent variable: without vs with metabolic syndrome.

# :Log transformation.

* Unfermented tea including green tea (unfermented) and oolong tea (partial fermented).

Nagelkerke R square = 0.526; Cox & Snell R square : 0.384.

**Table 5:** Binary Logistic Regression Model for Associated Factors (Focus on Total Smoking Amount and Duration of Habitual Tea Consumption) of Metabolic Syndrome in Elderly Males Living in a Rural Community (N = 361).

			Odds ratio	95%CI		*P*
Age			1.04	0.98,	1.10	0.17
Body mass index (kg/m^2^)			1.38	1.20,	1.58	<0.001
Uric acid (mg/dl)			1.44	1.16,	1.80	0.001
HOMA index#			4.65	2.58,	8.37	<0.001
hsCRP (mg/L)#			1.26	0.97,	1.64	0.09
Occupational status		(No = 0, Yes = 1)	0.78	0.39,	1.57	0.50
Lived with partner		(No = 0, Yes = 1)	1.71	0.76,	3.84	0.20
Literate		(No = 0, Yes = 1)	0.83	0.36,	1.92	0.67
Alcohol drinking		(No = 0, Yes = 1)	1.16	0.61,	2.22	0.65
Coffee drinking		(No = 0, Yes = 1)	0.86	0.25,	2.90	0.80
Habitual tea consumption						
(Non-current tea drinker = 0)			1			
(Tea drinking years<10 = 1)			0.36	0.15,	0.90	0.03
(Tea drinking years≥10 = 2)			0.55	0.28,	1.07	0.08
Total smoking amount (pack-year)						
		(Non-smoker = 0)	1			
		(0<Pack-years<30 = 1)	0.79	0.34,	1.84	0.59
	(Pack-years≥30 = 2)		2.78	1.31,	5.90	0.01
Physical activity (IPAQ-short form)						
		(Low = 0)	1			
		(Middle = 1)	0.67	0.31,	1.43	0.30
		(High = 2)	0.83	0.36,	1.92	0.66

HOMA: Homeostatic model assessment; hsCPR: high sensitivity C-reactive protein;

IPAQ: International Physical Activity Questionnaire.

Dependent variable: without vs with metabolic syndrome.

# :Log transformation.

Nagelkerke R square = 0.547; Cox & Snell R square : 0.400.

**Table 6:** Binary Logistic Regression Model for Associated Factors (Focus on Daily Smoking and Tea Consumption Amount) of Metabolic Syndrome in Elderly Males Living in a Rural Community (N = 361).

			Odds ratio	95%CI		*P*
Age			1.04	0.98,	1.10	0.20
Body mass index (kg/m^2^)			1.37	1.20,	1.57	<0.001
Uric acid (mg/dl)			1.40	1.13,	1.74	0.002
HOMA index#			4.66	2.59,	8.38	<0.001
hsCRP (mg/L)#			1.25	0.96,	1.63	0.10
Occupational status		(No = 0, Yes = 1)	0.80	0.40,	1.58	0.52
Lived with partner		(No = 0, Yes = 1)	1.54	0.69,	3.43	0.30
Literate		(No = 0, Yes = 1)	0.91	0.40,	2.06	0.82
Alcohol drinking		(No = 0, Yes = 1)	1.20	0.63,	2.27	0.58
Coffee drinking		(No = 0, Yes = 1)	0.94	0.28,	3.16	0.93
Daily tea consumption						
(Non-current tea drinker = 0)			1			
(Daily amount<240 cc = 1)			0.68	0.33,	1.39	0.29
(Daily amount≥240 cc = 2)			0.35	0.17,	0.72	<0.05
Daily smoking amount						
		(Non-smoker = 0)	1			
		(Daily amount<20 = 1)	1.00	0.41,	2.44	0.997
	(Daily amount ≥20 = 2)		2.54	1.24,	5.18	<0.05
Physical activity (IPAQ-short form)						
		(Low = 0)	1			
		(Middle = 1)	0.68	0.32,	1.43	0.30
		(High = 2)	0.81	0.35,	1.85	0.61

HOMA: Homeostatic model assessment; hsCPR: high sensitivity C-reactive protein;

IPAQ: International Physical Activity Questionnaire.

dependent variable: without vs with metabolic syndrome.

# :Log transformation.

Nagelkerke R square = 0.551; Cox & Snell R square : 0.403.

## Discussion

In this study, subjects who had a current smoking habit (especially those who smoke ≥20 cigarettes) had a significantly higher prevalence of MetS compared with non-smokers. Those who had a current tea drinking habit (especially those who consumed 240 cc daily) had a significantly lower prevalence of MetS compared with non-current tea drinkers. The results also showed evidence of cumulative dose-effects of smoking and tea drinking habits. To the best of our knowledge, this is the first community-based study that demonstrates the interrelationships among MetS, TSA and habitual tea consumption.

To identify previous related works, we used literature sources from Ovid MEDLINE with an English-language search from 1996 to December 2011 using the following search terms: aged, elderly, male, MetS, smoking habit, and tea drinking habit. The association between smoking habits and MetS is inconclusive in the literature, and this is probably due to differences in the various study designs or the races, ages and populations of the subjects [7], [20], [21]. Consistent with our findings, these studies concluded that a smoking habit ≥20 pack-years [22] and daily smoke amount ≥30 cigarettes [6] had positive association with MetS. It is plausible to suggest that not only current smokers but also the daily smoking amount and lifelong TSA are important associated factors for the development of MetS in the elderly. One study reported that quitting smoking is a stronger associated factor for incident of MetS than sustained smoking [23]. In contrast, few studies and ours showed no association between ex-smokers and MetS in comparing with non-smokers [6], [21]. The association of stopping smoking with MetS needs more study to reach a final conclusion.

Although the pathophysiology of MetS is not conclusive, there is evidence that insulin resistance is the common soil [24]–[26]. Smoking may induce insulin resistance and activate many inflammatory factors, which may lead to the MetS and CVD [27]. Studies show that many long term cigarette smokers are insulin resistant [28] and hyperinsulinemic [29], which may explain why MetS is more prevalent in current smokers or those with higher TSA in this study. After adjusting the HOMA index, our results still reveal a significantly positive association between current smokers, TSA and MetS, which highlights the complicated interrelationships among these factors, and this has important implications in clinical practice.

The negative association between tea drinking and MetS was clearly demonstrated, especially for current tea drinkers who drinking un- or partial fermented tea (green tea or oolong tea), for less than 10 years and more than 240 cc daily. Though data failed to show significant association for drinking tea for more than 10 years, but there was trend to have lower OR (*p* = 0.08) in association with MetS. The same result had been reported in another cross-sectional study in discussing the relationship between tea and hypertension [10]. Possible explanations would be due to: relative small study population or the survival effect which may interfere with the true association between tea consumption and MetS. Tea has several bioactive components, such as catechin, green tea polyphenol and epigallocatechin gallate (EGCG). Consistent consumption of 5 to 6 or more cups daily or 200 to 300 mg of EGCG has been shown to benefit cardiovascular and metabolic health [30].In addition, animal studies have suggested that both green and black tea suppress adipocyte differentiation, proliferation and fatty acid uptake into adipose tissue, as well as other important markers of MetS, such as serum TG, cholesterol, glucose, and insulin [31], [32], which may result from gene expression being regulated by tea [33]. In human studies, habitual tea consumption significantly reduces the risk of developing hypertension [10], and has a negative relation with body fat distribution and hyperglycemia [10]–[12] in the Chinese population. Although the mechanisms of tea with regard to its metabolic effects are varied and still not clearly understood, the protective role of tea drinking habit showed in our study might also be explained by the chemopreventive activity of EGCG by regulating multiple signaling pathways (e.g., VEGF, IGF-1 and EGFR) and kinase [34], [35].

Consistent with previous reports, our study further found a number of associated factors of MetS for elderly male Taiwanese, including hyperuricemia, higher BMI and HOMA indices. Many researchers have concluded the hyperuricemia is associated with the development of MetS [36], [37], and the significant relationship between UA and MetS may be mediated by visceral fat accumulation and hypoadiponectinemia [38], or be a consequence of obesity and dysregulation of the renin-angiotensin system [39]. Hyperuricemia may also promote inflammatory, ischemic or oxidative stresses, and increase the risk of CVD [40]. Clinicians should thus pay attention to the coexistence of MetS with hyperuricemia in order to control the associated consequences (high blood pressure, obesity, and so on.) in clinical practice [41].

Many studies have reported that BMI is significantly associated with MetS and is useful for predicting the onset of MetS and similar results were found in the current study [42], [43]. Evidence obtained from 30 years of follow-up revealed that men with MetS had increased risk of CVD and total death in all different BMI status [44]. These results show that BMI is a significant predictive factor for MetS and may increase the subsequent risk of CVD, as was also found in this paper. In our study, the insulin sensitivity reflected by the HOMA index was shown to be an independent predictor of MetS [22]. In obese subjects with MetS, the fact that the level of insulin resistance can be predicted by cigarette smoking may further emphasize the effect of cigarette smoking on this syndrome [45]. Although the predictive role of hs-CRP for insulin resistance and/or MetS had been disclosed in Han Chinese and Japanese populations [30], [46], [47], but no statistical significance in the current study.

Our study showed physical activity is significant associated with MetS only in univariate analysis. Studies reported inverse association between MetS and physical activity in the elderly [48]. There are studies focused on the elderly with the same result as our study. Previous study performed in Tainan city showed no significant association between physical activity and hypertension risk in the elderly [13]. An elderly community study performed in southern Brazil revealed similar odds ratio of having MetS for different physical activity categories [49]. The association between physical activity and MetS may be influenced by different types of activity [50]. The possible explanation may due to the majority of our study participants were farmers with higher mean physical activity load (median total physical activity were 5870 kcal/week) thereby can’t differentiated the effect of physical activity on MetS.

Our study has some potential limitations. First, the issue of recall bias may be significant, as it often appears with more complicated behavior or when something happened 20–30 years ago. However, for simple imprinted events, such as history of diseases, educational status and long-term life styles, the recall bias may be minimized [51]. Second, details of the daily diets of our participants was not gathered, although earlier research concluded that a cluster of multiple risk behaviors (e.g., low leisure-time physical activity, and low fruit/vegetable intake) are associated with higher levels of cigarette consumption [52]. Future studies should thus take all major behaviors and life style factors into consideration, as this would help to minimize possible confounding effects in the analysis. Although excess intake of energy, fat, or cholesterol may not be associated with a greater risk of MetS, especially for men [53], they should be considered in future studies. Third, it was arbitrary to use the 30 pack-years of smoking, 20 cigarettes daily, 10 years habitual tea drinking and 240 cc daily tea consumption to categorize the participants into different groups. However, the authors tested the hypothesis with different models and took all possible confounders into account, which all lead to universal conclusions. Fourth, our study population was aged over 65, and the survival effect thus cannot be overlooked when considering the results. Fifth, though we cannot fully excluded selection bias in this study but there was no statistically significant difference in mean age between responders and non-responders was found. This may indicated a relatively less biased result, but it should be interpreted in a relatively conservative manner. Finally, our study focused on elderly males living in a rural area with a high prevalence of MetS, and care should thus be taken when extrapolating the findings across a wider age range or more variety of population.

Further research may include an outcome study that focuses on the long term effects of habitual tea drinking and smoking habits on CVD and all cause mortality. Because the relationships among the key elements of tea drinking and smoking habits with MetS are still inconclusive, additional research is needed.

In conclusion, our study suggests that current smokers, especially those with a higher TSA and daily cigarette amount, are significantly positive associated with MetS. In contrast, current tea drinkers, especially for those subjects who had drunk tea for moderate duration and un- or partial fermented tea with more than 240 cc daily might have a negative association with MetS in elderly males dwelling in a rural community. As the growing epidemic of MetS and tobacco smoke exposure will synergistically increase the risk of CVD [54], anti-smoking campaigns and tea drinking for the elderly could be considered in facing the challenge of MetS.
